# GC-MS Analysis, Antibacterial and Antioxidant Potential of Ethyl Acetate Leaf Extract of *Senna singueana* (Delile) Grown in Kenya

**DOI:** 10.1155/2022/5436476

**Published:** 2022-08-18

**Authors:** Paul Ochieng Nyalo, George Isanda Omwenga, Mathew Piero Ngugi

**Affiliations:** ^1^Department of Biochemistry, Microbiology and Biotechnology, Kenyatta University, P.O Box 43844-00100, Nairobi, Kenya; ^2^Penda Health (K) Ltd, Medical Laboratory Department, P.O Box 22647-00100, Nairobi, Kenya

## Abstract

Bacterial diseases are a leading cause of mortality and morbidity globally. During bacterial diseases, an elevation of host immune response occurs, which involves the production of free radicals in response to the bacterial infection. The overproduction of free radicals in excess of the antioxidants leads to oxidative stress. Conventional antibiotics are linked to side effects such as hypersensitivity reactions in addition to bacterial pathogens developing resistance against them. Artificial antioxidants are said to be carcinogenic. This study sought to confirm folklore use and validate the antibacterial and antioxidant potential of *Senna singueana* which has been widely used in the Mbeere community. The *in vitro* antibacterial potentials of the plant extract were investigated on *Bacillus subtilis* ATCC 21332*, Escherichia coli* ATCC 25922, *Salmonella typhi* ATCC 1408, and *Staphylococcus aureus* ATCC 25923. Ciprofloxacin (100 *µ*g/ml) drug was used as a standard reference, whereas 5% DMSO was used as a negative reference. The antibacterial tests included disc diffusion and minimum inhibitory and bactericidal concentrations. *S. singueana* ethyl acetate extract showed broad-spectrum potential against tested bacterial microbes producing mean zones of inhibition (MZI) from 07.67 ± 0.33 to 17.67 ± 0.33 mm. The extract demonstrated a greater effect on Gram-positive than Gram-negative bacterial pathogens. Antibacterial properties of ciprofloxacin were significantly greater in comparison to plant extract in all the dilutions (*p* < 0.05), while 5% DMSO was inactive against all the tested bacteria. MBC values were greater than MIC values. Antioxidant properties of the extract were determined through scavenging effects of DPPH and hydroxyl radicals (^•^OH) as well as ferric reducing antioxidant potential (FRAP) assay. *S. singueana* demonstrated effects against all radicals formed. Additionally, the extract exhibited ferric reducing abilities. The extract also contained various phytocompounds with known antibacterial and antioxidant properties. This study recommends the therapeutic use of *S. singueana* as an antibacterial as well as an antioxidant agent.

## 1. Introduction

Since the ancient time, medicinal plants have been used to treat various infections. According to the World Health Organization (WHO), 80% of the global population uses a variety of plant fractions and their dynamic components as traditional therapies [1–4]. Phytochemicals produced by plants including tannins, phenolic compounds, and phytosterols have been demonstrated to have positive and significant benefits to human health [2]. Flavonoids and phenolic compounds, for example, have anticancer, antioxidant, antidiabetic, and anti-inflammatory potentials [5].

Medicinal plants are most commonly used in nonindustrialized and traditional cultures, owing to their ease of availability and lower cost when compared to modern medicines [6]. Many developing and developed countries use herbal medicine to maintain human well-being and personal health and treat bacterial diseases [7]. Medicinal plants are regularly used in rural societies where pharmaceuticals are unavailable or impossible to obtain. In contrast, in Westernized societies, medicinal plants are typically used as an alternative or supplement to prescribed medicine [8]. In Africa, for example, various communities have their unique approach to health and disease, even down to ethnopathogenic notions of diseases and therapeutic behavioural patterns [9].

In this study, we evaluated the antibacterial and antioxidant properties of the Kenyan grown *Senna singueana* medicinal plant which belongs to the family Fabaceae. The plant family Fabaceae includes legumes which are fruits of plants and plant is known as plant of bean or pea. Plants belonging to this family have various pharmacological benefits. For example, *Acacia catechu* which belongs to the *Acacia* genus in the Fabaceae family has traditionally been utilized to cure a variety of illnesses, particularly gastrointestinal as well as stomach-related issues [10]. Similarly, *Glycyrrhiza glabra* belonging to the *Glycyrrhiza* genus within this family has traditionally been used to treat a variety of bacterial ailments, including tonsillitis, sore throat, coughs, and diseases [5].

The genus *Senna* is categorized in the Fabaceae family, which contains over 300 species of tree shrubs and subshrubs that can be found in a variety of habitats and in continents like Africa, America as well as, to a lesser extent, Pacific islands and Asia [11]. *Senna* species have exhibited antimicrobial, anti-inflammatory, antidiabetic, and antimalarial properties [12]. *Senna* is widely used for a variety of purposes including decoration, building, nutrition, poisons, rituals, and medicine [11]. *Senna alata* bark decoction for example is used in East African communities to treat cuts after tribal mark incision and tattooing [12]. *Senna alata* leaves have been used in Thailand to treat wounds, constipation, and inflammation [13]; *Senna alexandrina* leaves and fruits have been used in Sudan to treat constipation and GIT disorders [14]; *Senna occidentalis* leaves, roots, and seeds have been used in India to treat respiratory diseases, malaria, diabetes, and urinary disorders [15, 16]; *Senna sophera* has been used to treat respiratory disorders in India [17]; and *Senna tora* has been utilized in China to treat liver illnesses, stomach disorders, and poor eyesight [18].

*S. singueana,* which is also known as scrambled egg, is a deciduous shrub with a light, open crown; it can grow to 1–15 meters tall. It has a spectacular flowering display which often takes place in the dry seasons [19]. It is an African traditional medicinal plant with many medicinal uses throughout the African continent [20, 21]. It is used as a therapy for diarrhea, conjunctivitis, bilharziasis, and coughs in different communities [20, 22]. The Mbeere community calls it Mukengeta and they use it to treat anthrax and elephantiasis [23]. It is also used in both humans and animals as a purgative and a lactation stimulant [24].

Previous studies on *S. singueana* reported several biological activities such as hepatoprotective and antiapoptotic properties of methanol bark extracts [21], antimalarial and antioxidant properties of ethanol leaf extracts [22, 25], antinociceptive effects for methanol leaf extracts [19], hypoglycemic capability of aqueous leaf extracts [26], and antibacterial activities of aqueous leaf extracts [27, 28]. Previous experiments have also demonstrated that both aqueous and methanol extracts of *S. singueana* are relatively nontoxic, and thus safe for use [29, 30]. It has been shown to have anticancer and antimalarial effects [25].

The plant has also been used traditionally to treat inflammatory conditions, convulsions, constipation, gonorrhea, and heartburn [31]. *S. singueana* leaves are used to treat a variety of poultry ailments in Zimbabwe, including coccidiosis, coughing, and flu-like illnesses [32]. The plant is also said to be used as food and fodder. Its leaves, pods, and seeds are fed to animals [33].

Bacterial pathogens and increase in antibacterial resistance have continued to rise, leaving patients with few or no alternative treatment options and an increase in diseases and deaths globally [34]. Bacterial infections cause almost half of human deaths in developing countries [35], a situation that may worsen due to misuse, overuse, or underuse of antibiotics leading to antibiotic resistance [36]. Globally, *Escherichia coli, Staphylococcus aureus,* and *Klebsiella pneumoniae* are the major causes of community and hospital-acquired bacterial diseases [37]. Bacterial infections also cause discomfort and suffering among infected individuals, thereby lowering their productivity [38].

Due to the lack of resources, infectious disease reports from developing countries are not well documented [39]. In Kenya, microbial pathogens that cause the majority of human diseases are generally those with high antibiotic resistance. The top five killers in Kenya are infectious diseases although the data on bacterial infections are not well documented because a majority of ailments and deaths occur outside the hospitals [40] with the high prevalence among Kenyans from poor communities [41].

During bacterial infections, the enzyme nicotinamide adenine dinucleotide phosphate (NADPH) oxidase catalyzes activated immune cells like neutrophils to undergo “respiratory burst” to produce ROS such as superoxide (O_2_^−^) [42]. Superoxide dismutase catalysis converts superoxide radicals to hydrogen peroxide (H_2_O_2_) [43], which is responsible for bacterial autolysis at the stationary phase [44]. Hydrogen peroxide can also be converted into a more toxic hypochlorous acid or react with superoxide to form hydroxyl radicals which in combination with the two ROS (hydrogen peroxide and superoxide) can kill any bacteria within the neutrophil [43]. As the bacterial infection persists in the body, more ROS as well as RNS are formed by activated immune cells. When these free radicals are produced in excess of available natural antioxidants, they contribute to oxidative stress leading to direct damage of cells [45].

Considering the adverse effects of the conventional antibacterial and antioxidant drugs, high cost, and the increased pathogenic resistance to these drugs, efforts have been and are being made to find alternative medicines from plants that are more effective with fewer side effects [9]. Traditional healers have indigenously used medicinal plants to cure several diseases including bacterial infections [46]. However, their indigenous uses lack scientific validation. The Mbeere community uses *S. singueana* as a remedy to cure a variety of diseases. This study sought to confirm its folklore use and validate antibacterial as well as antioxidant properties of ethyl acetate extract of *S. singueana*. This study also determined the phytochemical compositions of ethyl acetate extract of *S. singueana* to determine the basis of its medicinal potentials.

## 2. Materials and Methods

### 2.1. Plant Material Collection and Preparation

Fresh leaves of S. *singueana* were gathered from Gikuyari village, Thura Sub Location, in Embu County, Kenya in May 2021 with the assistance of a local practicing traditional herb doctor. GPS coordinates for the plant collection site are 0°35′39″ N and 37°38′12″ E. The plant sample was transported to Kenyatta University, where it was identified by a recognized taxonomist and a specimen preserved at Kenya National Museum's herbarium for future reference. Voucher number for the specimen was allocated as PN/001/27698/2018. The leaves were well washed using running tap water, rinsed using distilled water (DH_2_O), and chopped into small pieces. They were then shade dried for 28 days, finely ground into powder prior to storage at room temperature in airtight vessels ready for the extraction process.

### 2.2. Extraction Procedure

Four hundred grams of dry powder of *S. singueana* leaves were soaked in 1.2 L of ethyl acetate for 72 hours. The solution was occasionally swirled to achieve complete dissolution. After 72 hrs, the solution was decanted and vacuum filtered with the help of a Buchner funnel and Whatman's filter paper No. 1. Thereafter, using a rotary evaporator, the filtrate was concentrated to evaporate the solvent at 90 rpm at 60°C under vacuum. The extract yield of the plant was determined according to the following equation:

(1) $$\begin{array}{c} \text{Percentage Yield}=\left(\frac{K1}{K2}\right)\times 100\%, \end{array}$$

where K1 is the mass of concentrated plant extracts and K2 is the dry mass of the powdered plant before extraction [47].

The resultant extract was placed in airtight sterile clean glass containers and stored at 4°C awaiting bioassay studies.

### 2.3. Experimental Design

This study utilized a completely randomized study design.

### 2.4. Bacterial Test Organisms and Controls

Bacterial isolates used for antibacterial assays were sourced from Kenyatta University's Microbiology Laboratory, Biochemistry, Microbiology, and Biotechnology (BMB) Department. They comprised of *B. subtilis* ATCC 21332, *E. coli* ATCC 25922, *S. aureus* ATCC 25923, and *S. typhi* ATCC 1408. Ciprofloxacin was utilized as a positive reference (reference antibiotic), whereas DMSO (5%) was utilized as a negative reference.

#### 2.4.1. Maintenance of Bacterial Stock Cultures

To obtain fresh bacterial colonies, the bacterial stock pathogens were streaked on Mueller Hinton Agar (MHA) prior to 24 hours at 37°C [48]. Thereafter, 3 to 4 colonies were picked with a sterile wire loop and transferred in sterile glass tubes containing 10 ml of sterile Mueller Hinton Broth (MHB) followed by 24 hours of incubation at 37°C to obtain freshly grown bacterial suspensions which were kept at 4°C [48].

### 2.5. Preparation of Extract Concentrations and Disc Impregnation

One hundred (milligrams of *S. singueana* extract were weighed and then placed in a sterile 2-ml microcentrifuge tube. One milliliter of 5% DMSO was added, and the blend properly vortexed thereafter sonicated to ensure complete dissolution to achieve a 100 mg/ml stock solution concentration [49]. Twofold serially diluted dilutions were prepared by taking 500 *µ*l of extract's stock solution and mixing with 500 *µ*l of 5% DMSO to attain concentrations beginning from 50 mg/ml to 3.125 mg/ml. Fifteen (microliters of serially diluted extract was used to impregnate sterile discs. The discs were left in the biosafety cabinet to air dry for about 20 minutes before being placed on the surface of inoculated media. Ciprofloxacin powder (100 *µ*g) dissolved in 1000 *µ*l of sterile normal saline [50] was applied as the positive reference, whereas DMSO (5%) was applied as the negative reference.

### 2.6. Antibacterial Sensitivity Tests

Antibacterial sensitivity assays were conducted using the disc diffusion technique in triplicates as explained by Benkova et al. [51] and Wolde et al. [52]. Sterile cotton swabs were dipped in the bacterial inocula and rotated on the tube's sides to eliminate surplus fluid. After which, they were streaked all over the already prepared Mueller Hinton Agar media. To guarantee the inocula's even distribution, the plates were rotated approximately 60 degrees each time. The inoculated plates were then left to dry for about 5 minutes in a biosafety cabinet before placing the discs on the surface. Using sterile forceps, the 6-mm paper discs impregnated with various dilutions of *S. singueana* extract, 5% DMSO (negative reference), and Ciprofloxacin (positive control) were then placed on the agar surface, one at a time. The plates were placed in sterile condition at normal room temperature (RTP) for around 15 mins to allow for infiltration of the extract, 5% DMSO, and Ciprofloxacin into the Mueller Hinton Agar media, then incubated at 37^o^C for 24 hours [53], after which the clear zones around the discs were determined in millimeters (mm) using a ruler and recorded in spreadsheets. Based on criteria detailed by Mwitari et al. [54], the antibacterial potential of the studied extract and the positive control was determined as follows;

- Zones of inhibition <7 mm were considered not to have any activity,
- Zones between 8 and 11 mm were considered active, and
- Zones >11 mm were considered very active.

### 2.7. Minimum Inhibitory Concentration (MIC)

To determine the minimum inhibitory concentration, a broth dilution experiment was done in triplicates following the protocols as performed by Manandhar et al. [53]. The extract was double diluted to concentrations (conc) from 100 mg/ml to 1.5625 mg/ml in sterile 96-well plates containing MHB. This was done by adding equal volumes (100 *µ*l) of the extract to MHB. After dilution, 20 *µ*l of each test bacterial suspension, adjusted to standard turbidity (0.5 McFarland), was pipetted to the wells prior to 24-hour 37°C incubation. Finally, 1% of resazurin solution (50 *µ*l) was added to every well as an indicator. Thereafter, the plates were re-incubated at 37°C for 30 mins [53]. The minimum concentration that inhibited visible blue to pink resazurin color change was considered minimum inhibitory concentration [55]. Ciprofloxacin powder (100 *µ*g/ml) was diluted the same way the extracts were diluted, while 5% DMSO was used as a negative reference.

### 2.8. Minimum Bactericidal Concentrations (MBCs)

Using a sterile cotton swab, 10 *μ*l of the materials from every well having concentrations at and above the MICs of studied antibacterial agents was spread all over the surface of the MHA plate followed by 37°C incubation for 24 hours [48]. MBC was documented as the minimal concentration with no visible bacterial growth on MHA [56]. Bacterial growth on the MHA plates was recorded as bacteriostatic effects of the extracts, whereas a lack of bacterial growth on the MHA plates was considered as bactericidal effects of the investigated extracts. This was done in triplicates.

### 2.9. Determination of *In Vitro* Antioxidant Activities

#### 2.9.1. *In Vitro* DPPH Radical Scavenging Capability

*S. singueana* extracts' ability to mop 2, 2-diphenyl-1-picrylhydrazyl (DPPH) radicals was done in triplicates as conducted by Arika et al. [57], with little modifications. The plant extract and ascorbic acid (reference) were prepared at various concentrations beginning from 15.625 *µ*g/ml to 500 *µ*g/ml. DPPH (1 mM) solution was prepared in methanol. One milliliter of each dilution of the test extract and the standard was separately placed in clean test tubes, after which DPPH (0.5 ml) and methanol (3 ml) solutions were added. The blend was thoroughly vortexed for 5 minutes; thereafter, it was set aside in a dark cupboard for 30 mins at ambient temperature. A blank solution containing 3 ml methanol and 0.5 ml DPPH solutions was also prepared. Using a spectrophotometer, the solutions' absorbances were measured at 517 nm against blank. The plant extracts' % DPPH free radical quenching properties were computed as

(2) $$\begin{array}{c} \%\;DPPH\text{ Radical Scavenging activity}=\frac{\text{Abs Blank}-\text{Abs Sample}}{\text{Abs Blank}}\times 100. \end{array}$$

Half maximal inhibitory concentration (IC_50_), representing the concentration at which 50% of the DPPH radicals were mopped, was analyzed using linear regression analysis [58].

#### 2.9.2. Determination of Ferric Reducing Antioxidant Capacity

Plant extracts' ferric reducing potential was determined following the protocol used by Park et al. [59], with little modifications. Approximately 2.5 ml solution of test extracts and reference (Ascorbic acid) at varying concentrations starting from 7.8125 *µ*g/ml to 500 *µ*g/ml were separately blended with 2.5 ml phosphate buffer (pH 6.6, 0.2 M) along with 2.5 ml potassium ferricyanide (1%) followed by 20 minutes incubation at 50^o^C. Thereafter, 2.5 ml of 10% trichloroacetic acid was added into the blend and vortexed before being centrifuged for 10 minutes at 3000 rotations per min (rpm). The supernate (5 ml), DH_2_O (5 ml), and 0.1% ferric chloride (1 ml) were mixed, incubated at normal room temperature (RTP) for 10 minutes, after which a spectrophotometer set at 700 nm was used to read the absorbance against blank. Blank solution comprised all the reagents other than the plant extract and ascorbic acid. This was done in triplicates.

#### 2.9.3. Determination of *In Vitro* Hydroxyl Radical Scavenging Effects

The extracts' ability to quench hydroxyl radicals was done based on protocols carried out by Arika et al. [57] and Zhang et al. [60]. A blend solution of extract/control (Gallic Acid) at varied dilutions (15.625 *µ*g/ml to 500 *µ*g/ml) 500 *µ*l, 28.0 mM 2-deoxy-2-ribose dissolved in KH_2_ PO_4_–KOH buffered solution (20.0 mM, pH 7.4) 100 *µ*l, EDTA (1.04 mmol L^−1^) 100 *µ*l, ascorbic acid (1.0 *µ*M) 100 *µ*l, FeCl_3_ (200 mM) 100 *µ*l, and hydrogen peroxide (1.0 mM) 100 *µ*l. This followed incubation of the blend at 37°C in a water bath for 60 minutes then 1% cold thiobarbituric Acid (TBA) 1000 *µ*l along with 2.8% trichloroacetic Acid (TCA) 1000 *µ*l solutions were added before heating the blend at 100°C for 15 minutes where a noticeable pink color developed; thereafter, the mixture was cooled in cold water. Absorbance was recorded against blank using a spectrophotometer set at 532 nm. Assays were run in triplicates. The % radical quenching capability was computed as [57]

(3) $$\begin{array}{c} \%\text{Hydroxyl Radical Scavenging activity}=\frac{Abs\text{ Blank}-\text{Abs Sample}}{Abs\text{ Blank}}\times 100. \end{array}$$

### 2.10. Quantitative Phytochemical Activities

A clean microcentrifuge tube (2.0 ml) was labeled as SS for *S. singueana*. To the labeled tube, 1 mg of the test extract was added followed by 1000 *µ*l of ethyl acetate to liquefy the sample. The sample was vortexed for 1 min then sonicated for 15 minutes after which it was centrifuged at 1,400 rotations per min for 5 mins. The resultant supernate (1 mg/ml) dried over anhydrous Na_2_SO_4_ was used to prepare experimental solutions in triplicates at a concentration of 100 ng/*µ*L [57].

GC-MS was done on 7890 A Gas-Chromatograph joined to a 5975°C mass selective detector (Agilent Technologies), which consists of an HP-5 MS low bleed capillary column (30 m long, 0.25 mm wide, as well as 0.25 *μ*m film thick). Operating parameters of the mass spectrometer included: relative detector gain mode, 70 eV of ionization energy, 3.3 mins of filament delay time, 1666*μ*/sec of scan speed, 40–550 m/*z* of scan range, 230 C ion source temp, and 180°C quadrupole temp. Helium gas (99.9%) was applied as a carrier gas at a steady flow speed rate of 1.25 ml per min. Mass transfer temperature was programmed at 200°C while injector line transfer temperature was programmed at 250°C, with 1 *μ*l injection volume. The oven temperature was programmed at 35°C for 5 mins followed by a 10°C/min increase to 280°C for 24.5 mins and then raised at a rate of 50°C per min to 285°C for 20.5 mins and a total run period of 50 mins. To identify the phytocompounds found in the extract, a comparison of the obtained data was matched with mass-spectral library search reports from the National Institute of Standards and Technology (NIST) 08 and 11, where each unique peak represented a particular chemical substance.

### 2.11. Data Management and Statistical Analysis

This study's data were tabulated in a Microsoft Excel spreadsheet and organized before being imported into Minitab software version 17.00, where descriptive statistics values were conveyed as mean ± STD (standard) error of mean (SEM). One*-*way analysis of variance (ANOVA) for inferential statistics and Tukey's post hoc test for pairwise comparison as well as separation of means were used. A statistically significant *p* value of <0.05 was used. Comparison of the plant extract and standard antibacterial and antioxidant properties were done using unpaired Student's *T-*test. Graphs and tables were used to present the findings.

For GC-MS data, the various compounds were recognized primarily on their retention time (RT) and fragmentation pattern in comparison with the NIST 08, 11 library search report. For identification of the compounds, an identity match of above 60% with the library phytocompounds was required. The compound names, molecular weights, and structures were established. The components' concentrations were expressed in *µ*g/g.

## 3. Results

### 3.1. Yield of the Plant Extract

The percentage yield of the extract was 4.99%, producing a dark green solid extract.

### 3.2. Antibacterial Sensitivity of the Extract

**Figure 1:**
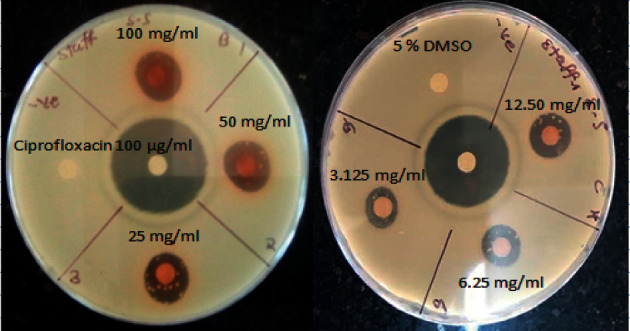
Inhibition zones caused by *Senna singueana* extract on *S. aureus*.

*S. singueana* extract exhibited notable antibacterial effects against all the tested bacteria in this study. This was manifested by the visible inhibitory zones surrounding the paper discs impregnated with various dilutions of the extract (Figure 1).

Generally, *S. singueana* extract demonstrated a greater potential effect against tested Gram-positive (+ve) pathogens (*B. subtilis* and *S. aureus*), recording greater than (>) 12 mm of mean zones of inhibition (MZI) at extract concentration ranges from 25.00 mg/ml to 100.00 mg/ml. At 12.5 mg/ml concentration, the extract had a MZI >12 mm on *S. aureus* (Table 1). The extract showed activity at all tested concentrations against the tested Gram-positive pathogens. However, *S. singueana* extract displayed no effect against tested Gram-negative (-ve) pathogens at concentration ranging from 3.125 mg/ml to 12.5 mg/ml (Table 1).

At 100 mg/ml concentration, *S. singueana* extract displayed antibacterial activities against both *S. aureus* and *B. subtilis* that were significantly different from the activities recorded by Ciprofloxacin, DMSO, and extract concentrations ranging from 3.125 mg/ml to 50.00 mg/ml (*p* < 0.05; Table 1). *S. singueana* extract's concentration at 100 mg/ml recorded MZI on *E. coli* and *S. typhi* that was statistically similar to 50 mg/ml concentration (*p* < 0.05; Table 1) but significantly distinct from concentration ranging from 3.125 mg/ml to 25 mg/ml (*p* < 0.05; Table 1).

*S. singueana* extract at 50 mg/ml concentration showed activities against all pathogens tested, although the activities were not significantly distinct from the activities of extract concentration 25 mg/ml on *E. coli* and *S*. *aureus* (*p* < 0.05; Table 1). Similarly, *S. singueana* extract effect at concentration 25 mg/ml was significantly different from activities of extract concentrations ranging from 3.125 mg/ml to 12.5 mg/ml against all pathogens except against S. *aureus* whose activity was statistically similar with concentration 12.5 mg/ml (*p* < 0.05; Table 1). At concentration 12.5 mg/ml, *S. singueana* extract had antibacterial effects against *S. aureus* and *B. subtilis* pathogens only even though the effects were not significantly different from activities of concentration 6.25 mg/ml (*p* < 0.05; Table 1). Similar activities were seen with concentration 3.125 mg/ml, whose effect was statistically similar to that of 6.25 mg/ml (*p* < 0.05; Table 1).

*In vitro* antibacterial potential of S. *singueana* extract was dose dependent with recorded MZI against all the tested bacterial pathogens, increasing with an increase in extract concentrations (Table 1). Dimethylsulfoxide (DMSO) was inactive on all the tested bacterial pathogens (MZI 6 mm). Ciprofloxacin (standard antibiotic) demonstrated significantly higher antibacterial activity against all studied pathogens producing significantly larger zones of bacterial growth inhibition than all extract concentrations used in the study (*p* < 0.05; Table 1).

### 3.3. Minimum Inhibitory Concentrations

The tested extract showed bacterial growth inhibitions against all tested bacterial pathogens and thus subjected to MIC and MBC tests. The mean MIC means ranged from 1.30 ± 0.26 to 20.83 ± 4.17 mg/ml (Table 2). *S. singueana* extract showed statistically similar inhibitory properties on *B. subtilis*, *S. aureus*, and *E. coli* (*p* < 0.05; Table 2). Similar activity was seen in the inhibitory effects of *S. singueana* extract on *S. typhi* and *E. coli* (Table 2). Ciprofloxacin demonstrated statistically similar inhibitory effects against all the tested pathogens (*p* < 0.05; Table 2).

In comparison to Ciprofloxacin, the ethyl acetate leaf extract of *S. singueana* exhibited inhibitory effects at significantly higher concentrations than Ciprofloxacin on all the tested bacterial pathogens (*p* < 0.05; Table 3).

### 3.4. Minimum Bactericidal Concentrations (MBCs)

Generally, the tested extract had higher MBC values than MIC values against each of the tested bacterial pathogens (Tables 2 and 4). Mean MBC ranged from 12.50 ± 0.00 to 100.00 ± mg/ml (Table 4). The ethyl acetate extract of *S. singueana* exhibited significantly higher bactericidal effects against *S. aureus* than *B. subtilis* (*p* < 0.05; Table 4). However, its bactericidal effects on *S. typhi* and *E. coli* were statistically similar (*p* < 0.05; Table 4). Ciprofloxacin demonstrated statistically similar bactericidal effects on *S. aureus*, *B. subtilis*, and *S. typhi* (*p* < 0.05; Table 4).

In comparison to Ciprofloxacin, the bactericidal effects of *S. singueana* extract against all tested bacterial pathogens were at significantly higher concentrations than Ciprofloxacin (*p* < 0.05; Table 5).

### 3.5. *In Vitro* Antioxidant Properties of *S. singueana* Ethyl Acetate Extract

#### 3.5.1. *In Vitro* DPPH Radical Scavenging Properties of *S. singueana* Extract

The tested plant extract displayed DPPH radical scavenging effect across all concentrations in a dose-dependent trend. As plant extracts' concentration decreased, DPPH radical scavenging capacity also decreased (Figure 1). S. *singueana* extract and ascorbic acid each showed significantly different DPPH radical quenching properties at all the dilutions (*p* < 0.05; Figure 1) except at concentrations between 250.00 *µ*g/ml and 500.00 *µ*g/ml where they each exhibited statistically similar DPPH free radical scavenging effects (*p* < 0.05; Figure 1). The reference, ascorbic acid, demonstrated a significantly greater scavenging potential of DPPH radicals with an IC_50_ value of 20.54 ± 2.24 *µ*g/ml in comparison to the studied plant extract which had an IC_50_ value of 47.97 ± 0.69 *µ*g/ml (*p* < 0.05).

#### 3.5.2. *In Vitro* Ferric Reducing Antioxidant Potential of Ethyl Acetate Extract of S. *singueana*

**Figure 2:**
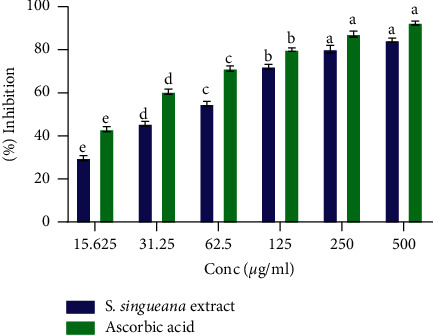
*In vitro* DPPH radical quenching properties of *S. singueana* extract. Bar graphs having identical letters across the tested concentrations are statistically similar (*p* < 0.05) (one-way ANOVA and Tukey's post hoc tests). Within the same concentration, bar graphs without asterisks (^*∗*^) are significantly distinct (*p* < 0.05) (two-Sample *T*-Test).

The seven tested dilutions of ethyl acetate leaf extracts of S. *singueana* showed dilution-dependent ferric reducing potential. All the extract concentrations exhibited a significantly lower ferric reducing activity than ascorbic acid (reference control) (*p* < 0.05; Figure 2) except at the lowest concentration of 7.8125 *µ*g/ml where the ferric reducing potential of the extract exhibited statistical similarity to that of reference (ascorbic acid) (*p* < 0.05; Figure 2). The ferric reducing capability of ascorbic acid's concentrations were significantly distinct, with the highest concentration exhibiting the highest effect (*p* < 0.05; Figure 2). However, S. *singueana* extract showed statistical similarity in ferric reducing activity at concentrations ranging from 7.8125 *µ*g/ml to 31.25 *µ*g/ml and concentration between 125 *µ*g/ml and 250 *µ*g/ml (*p* > 0.05; Figure 2), although the effect of extract concentration of 500 *µ*g/ml was significantly greater than all the other concentrations (*p* < 0.05; Figure 2).

#### 3.5.3. *In Vitro* Hydroxyl (^•^OH) Radical Scavenging Potential of Ethyl Acetate Extract of *S. singueana*

**Figure 3:**
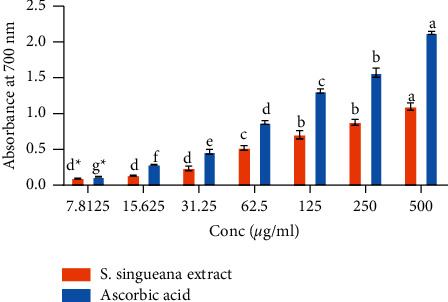
Ferric reducing potential of *S. singueana* extract. Bar graphs with identical letter/s across the tested concentrations are statistically similar (*p* > 0.05) (one*-*way ANOVA and Tukey's post hoc tests). Within the same concentration, bar graphs without asterisks (^*∗*^) are significantly distinct (*p* < 0.05) (two-sample *T*-Test).

The studied plant extract displayed an efficient hydroxyl free radical scavenging capability which occurred in a dilution-dependent trend (Figure 3). As illustrated in Figure 3, the hydroxyl (^•^OH) radical scavenging potential of gallic acid was significantly greater than that of *S. singueana* extract in all the tested dilutions (*p* < 0.05; Figure 3). There was a significantly different hydroxyl radical scavenging activity among all the tested concentrations of S. *singueana* extract (*p* < 0.05; Figure 3). As the extracts' concentrations decreased, its ^•^OH radicals' quenching capability also decreased with the lowest concentration demonstrating significantly the lowest effect (*p* < 0.05; Figure 3). Additionally, our findings showed that gallic acid had a significantly lower IC_50_ value of 35.33 ± 0.88 *µ*g/ml as compared to the extract whose IC_50_ value was 67.84 ± 1.34 *µ*g/ml (*p* < 0.05), indicating that gallic acid had a greater hydroxyl radical scavenging effect than the extract.

### 3.6. Quantitative Phytochemical Composition of Ethyl Acetate Extract of *S. singueana*

The S. *singueana* extract displayed the presence of a total of 51 compounds, out of which 33 compounds have known biological activities. Based on the obtained results, oxazolidine, 2-ethyl-2-methyl-, an Oxazoline compound, had the least concentration of 0.02 ± 0.00 *µ*g/g, whereas Squalene, a triterpenoid, had the highest concentration of 5.24 ± 0.07 *µ*g/g (Table 6). The findings also revealed a composition of 51.84% hydrocarbons, 24.3% terpenoids, 15.18% fatty acids, 3.26% tocopherols, 3.33% phenolic compounds, 1.03% iodo compounds, 0.28% steroids, 0.28% benzene derivatives, 0.09% heteroaromatic molecules, 0.09% volatile organic compounds, 0.07% cyclic secondary amines, 0.15% aminopyridine, 0.05% alkaloids, and 0.03% oxazoline compounds.

## 4. Discussion

Traditional plants produce natural products that have been known to be effective against bacterial infections, with few side effects compared to commercial antibiotics [61]. Many plants have been traditionally used to cure bacterial diseases; however, they lack scientific validation and documentation on their usage. This study evaluated the *in vitro* antibacterial properties of ethyl acetate extract of the Kenyan grown *S. singueana* on *B. subtilis*, *S. typhi*, *E. coli*, and *S. aureus*.

S. *singueana* extract exhibited antibacterial potentials on the tested Gram-positive and Gram-negative bacterial pathogens. The mean zones of inhibition (MZI) recorded against all the studied bacterial pathogens were dependent on the extract concentrations (zones decreased with a decrease in extracts concentration). This is in agreement with Adedoyin et al. [62], who demonstrated that the essential oil of *S. singueana* flowers had antibacterial properties in a dose-dependent manner. Our findings also agree with a study by Jambwa et al. [24], which demonstrated that the ethyl acetate fraction isolated from *S. singueana* leaves crude extract had antibacterial effects on both Gram-positive (*S. aureus*) and Gram-negative (*Salmonella Enteritidis* and *E. coli*) bacterial pathogens tested.

In this study, *S. singueana* extract inhibited the bacterial growth of the tested pathogens, producing MZI ranging from 07.67 ± 0.33 to 17.67 ± 0.33 mm with higher effects against Gram-positive bacterial pathogens. This is in consensus with a past report which illustrated that the methanol, acetone, and chloroform root extracts of *S. singueana* had greater activities on Gram-positive (+ve) bacteria (*Streptococcus pyogenes, S. aureus*, and *Streptococcus pneumonia*) than Gram-negative (−ve) pathogens (*Pseudomonas aeruginosa, S. typhi, E. coli,* and *Klebsiella pneumonia*) [63]. This also concurs with a report by Kareru et al. [27], which showed that the aqueous leaf extracts of *S. singueana* had higher effects on *S. aureus* and *B. subtilis* than it had on *E. coli*. This is also in agreement with Jibril et al. [64] who demonstrated the broad-spectrum antibacterial effects of methanol and ethyl acetate leaf extracts of *S. singueana*. However, our findings are partly contrary to the reports of Shawa et al. [20], which suggested that aqueous leaf and root extracts of *S. singueana* were inactive on *S. aureus* and *Pseudomonas aeruginosa*.

Gram-positive microbes are more susceptible to antibacterial agents [65], thus making them more sensitive to crude plant extracts and bioactive constituents. This could be the possible explanation as to why the studied extract had higher activities on Gram-positive bacteria pathogens.

The antibacterial potentials of the studied plant extract in this experiment are ascribable to the presence of various phytocompounds like terpenoids, alkaloids, fatty acids, hydrocarbons, phytosterols, and phenolic compounds. This concurs with a past study that showed the presence of such compounds in the roots, leaves, and seeds of *S. singueana* [21]. This is also in accordance with a study by Kolawole et al. [31], which demonstrated the presence of phenols, terpenoids, steroids, and alkaloids in ethanolic leaf extract of *S. singueana*.

Compounds like terpenoids have known antibacterial potentials [66]. They interfere with bacterial oxygen uptake and oxidative phosphorylation which are two important essential processes in bacteria [67]. Squalene, a triterpenoid, which had the highest concentration in the studied plant extract, has been shown to exhibit antibacterial effects [31]. Phytol, a diterpenoid, also present in the extract has known antibacterial activity [68]. Similarly, phytol acetate<E->, another diterpenoids, has also been shown to have antibacterial effects [69].

Majority of the hydrocarbons found in this extract were alkanes. Alkanes act by interfering with bacterial cell membrane integrity and function leading to bacterial cell death [70]. Octacosane, a straight-chain alkane present in the studied extract has known antibacterial properties [71]. Additionally, tetracosane and tricosane, other straight-chain alkanes, have also demonstrated antibacterial effects [72, 73].

Many plants utilize fatty acids in defense against pathogenic bacteria. Their prime target is disrupting the electron transport chain of bacterial cell membranes. Fatty acids can also act by inhibiting bacterial enzyme activity, impairment of nutrient uptake, and direct bacterial cell lysis [74]. Fatty acid like Hexadecanoic acid ethyl ester, present in this extract, has been found to possess antimicrobial capabilities [66]. Similarly, lauric acid, another fatty acid, has also previously been demonstrated to have antibacterial potential [75].

Alkaloids, a structurally diverse group of plant secondary metabolites, exert their antibacterial activity by inhibiting bacterial enzyme activity as well as causing disruption of the bacterial membrane thus killing the bacteria [76]. An alkaloid 2-cyclohexylpiperidine which was present in the studied extract has been known to have antimicrobial activities [77].

Phenolic compounds, large heterogeneous secondary plant metabolites, are known for their cell lysis in addition to membrane-disturbing capabilities as their mode of antibacterial activity [78]. Methyleugenol, a phenolic compound, present in the extract, has been shown to have antibacterial activities [79].

Phytosterols stabilize plant cell phospholipid bilayers just like cholesterols in animal cell membranes [80]. They have a resemblance to sterols which are found in the bacterial cells; thus, the phytosterols replace the normal sterols in the bacterial cell membrane, thus disrupting the bacterial cell membrane hence killing the bacteria [81]. *β*-Sitosterol, which was present in the extract, has been found to exhibit antimicrobial activities [82].

Vitamin E (*α* tocopherol) confers its antibacterial potential by acting as an antibacterial adjuvant in combination with other antibacterial agents [83]. Vitamin E, which was present in *S. singueana* extract, has been shown to exhibit antimicrobial activities [84]. Other compounds like fatty alcohols previously demonstrated antibacterial potentials [85]. A previous experiment by Malarvizhi et al. [86] confirmed that 3,7,11,15-tetramethyl-2-hexadecen-1-ol, a terpene alcohol, has been demonstrated to have antibacterial effects.

The high availability of free radicals in excess of antioxidants results in oxidative stress [87], which leads to cellular impairment and oxidative stress (OS)-related diseases [88]. To reduce the effects of oxidative stress in cells, antioxidants are produced, which counteracts the upshot of unstable free radicals by either reacting with them or neutralizing them by donating electrons to stabilize them [57]. Curative plants have for long been utilized to manage illnesses as a result of oxidative stress. The plant extract studied in this study showed potent *in vitro* antioxidant capabilities.

In this study, plant extract's and standards' antioxidant capacities in all the assays were in a dose-dependent trend. As the extract's/standards' concentration decreased, the antioxidant capacity also decreased. These findings concur with a previous study that demonstrated dose-dependent antioxidant potentials of ethyl acetate, petroleum ether, and methanol root bark extracts of S. *singueana* [89].

DPPH radical scavenging method is the most popular *in vitro* antioxidant method because it is easy, accurate, more sensitive, and more economical, whose outcome is highly reproducible as well as easily comparable with other free radical scavenging assays. In this method, when antioxidants in the tested extract react with DPPH, the DPPH accepts hydrogen atoms from the antioxidant making it lose its color from purple to yellow in a concentration-dependent manner measured at 517 nm [90].

In this experiment, the reference, ascorbic acid, had a greater scavenging capacity of DPPH radicals with IC_50_ value of 20.54 ± 2.24 *µ*g/ml in comparison to the plant extract, which had an IC_50_ value of 47.97 ± 0.69 *µ*g/ml. This is in agreement with the findings of Jambwa et al. [24], which demonstrated that the ethyl acetate fraction isolated from *S. singueana* leaves crude extract exhibited DPPH radical scavenging potential but with a lower IC_50_ in comparison to the standard ascorbic.

Our findings also concur with Hilawea et al. [89], who demonstrated that DPPH radical scavenging ability of ethyl acetate, petroleum ether, and methanol root bark extracts of *S.* s*ingueana* and ascorbic acid followed a similar dose-dependent trend. The researchers also found out that ascorbic acid had a greater DPPH radical scavenging activity than ethyl acetate, petroleum ether, and methanol root bark extracts of *S. singueana*.

The ferric reducing power of a substance depends on its ability to convert Fe^3+^ (ferric) to Fe^2+^ (ferrous) complex forming a Prussian blue-colored solution, with a directly comparative intensity to the substance's concentration. A greater absorbance read at 700 nm indicates a higher reducing capability of the substance [57]. Ferric reducing power results in this study demonstrated that vitamin C had significantly higher reducing power than the studied plant extract. Our findings concur with Hilawea et al. [89], who confirmed that the reducing capacity of ethyl acetate, petroleum ether, and methanol root bark extracts of *S. singueana* decreased with a decrease in extracts concentration. Additionally, they also noted that ascorbic acid had a greater reducing activity than the S. sin*gueana* extracts studied.

The extract's potential to scavenge hydroxyl radicals utilizes the principle that the extract will hinder ^•^OH radical-mediated deoxyribose deterioration via Fenton's reaction using Fe^3+^ + EDTA + ascorbic acid + hydrogen peroxide reaction blend [57]. Among the free radicals, hydroxyl radicals are considered extremely reactive and the most harmful free radical as its interaction with the cell membranes can damage sugar groups and the DNA base pairs leading to cell death and eventually mutation, which might also cause cancer, aging, and other chronic-related diseases [91].

Findings of this study confirm that the plant extract demonstrated significant hydroxyl radical scavenging activities. However, these findings showed that the standard had greater hydroxyl radical scavenging capacity in comparison to the studied extract. This is in agreement with a previous study by Gerezgher et al. [25], who found that the standard had a higher hydroxyl radical scavenging activity than the ethanolic leaf extract of *S. singueana*.

Antioxidant activities exhibited by S. s*ingueana* extract could be attributed to the availability of various phytocompounds that work synergistically to overcome free radicals [92]. Several biological compounds including phenolic compounds; lipids like fatty acids, phytosterols, and fatty acid esters; terpenoids like monoterpene, diterpenes, and triterpenes; alkaloids; and hydrocarbons like alkanes and alkenes were detected. They have been shown to exert their antioxidant capability through multi-step processes that involve initiating propagating and eventually terminating free radicals [92].

Terpenoids are known to have antioxidant activities. Terpenoids act as antioxidants by scavenging free radicals [93], through the donation of hydrogen to free radicals to stabilize them [94]. They also act as chelating agents [95]. A 7-octenal,3,7-dimethyl-monoterpenoid, present in *S.* singu*eana* extract has been known to have antioxidant effects [96]. Squalene, a triterpenoid, has also been shown to possess antioxidant effects [31].

Fatty acids exert antioxidant activity by scavenging free radicals [97]. Dodecanoic acid, a fatty acid, present in the extract, has previously been demonstrated to have antioxidant properties [98]. Ethyl hexadecanoate acid ethyl ester, another fatty acid, which was also present in the studied extract, has been shown in previous studies to have antioxidant activities [99].

Phenolic substances protect against free radicals by donating hydrogen atoms or electrons to unstable radicals [100] and chelating metal cations [101]. Methyleugenol, a phenolic compound, present in *S. singueana* extract is known to have antioxidant properties [102].

Phytosterols exert their antioxidant properties through the donation of electrons to unstable free radicals to make them stable [103]. *β*-Sitosterol, which was present in *S. singueana* extract, has been found to have antioxidant effects [104].

Tetratetracontane, a hydrocarbon present in *S. singueana* extract, has been shown to have antioxidant activities [105]. Tetracosane, another hydrocarbon also present in the studied extract, has also been shown to exhibit antioxidant potentials [106]. Other compounds like vitamin E, which was present in the studied extract, has been confirmed to possess antioxidant potentials [84].

The purpose of this study was to validate the traditional use of the leaves of *S. singueana* medicinal plant against common bacteria that cause several human infections, such as *E. coli, Bacillus subtilis, Salmonella typhi,* and *Staphylococcus aureus*, by evaluating its *in vitro* antibacterial and antioxidant properties and the presence of phytochemicals with such activities.

## 5. Conclusions and Recommendations

The findings of this study give a basis for the utilization of *S. singueana* in the treatment of bacterial infections and oxidative stress related infections. The extract also showed the presence of several phytocompounds that could be used in developing new antibacterial and antioxidant agents. The fatty acids, terpenoids, phenols, and others in this extract justify the obtained results.

## Figures and Tables

**Figure 4:**
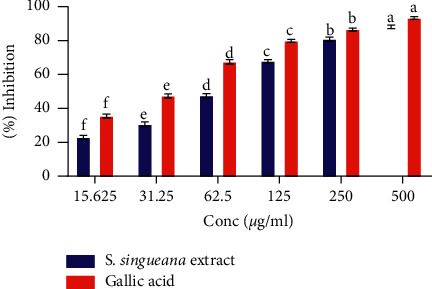
*In vitro* ^•^OH radical quenching potential of ethyl acetate leaf extract of *S. singueana.* Across the tested concentrations, bar graphs with identical letter/s are statistically similar (*p* > 0.05) (one-way ANOVA and Tukey's post hoc tests). Within the same concentration, bar graphs without asterisks (^*∗*^) are significantly distinct (*p* < 0.05) (two-Sample *T*-Test).

**Table 1:** Antibacterial properties of ethyl acetate leaf extracts of *S. singueana.*

Treatment	Zones of inhibition (mm)			
5% DMSO	*S. aureus*	*B. subtilis*	*E. coli*	*S. typhi*
Ciprofloxacin (100 *µ*g/ml)	06.00 ± 0.00^g^	06.00 ± 0.00^g^	06.00 ± 0.00^d^	06.00 ± 0.00^d^
*S. singueana* extract	26.33 ± 0.33^a^	29.67 ± 0.33^a^	32.67 ± ±0.33^a^	28.33 ± 0.33^a^
(mg/ml)				
100	17.67 ± 0.33^b^	17.67 ± 0.33^b^	09.67 ± 0.33^b^	10.67 ± 0.33^b^
50	15.33 ± 0.33^c^	14.33 ± 0.33^c^	08.67 ± 0.33^bc^	09.67 ± 0.33^b^
25	13.67 ± 0.33^cd^	12.33 ± 0.33^d^	07.67 ± 0.33^c^	07.67 ± 0.33^c^
12.5	12.67 ± 0.33^de^	10.67 ± 0.33^e^	06.00 ± 0.00^d^	06.00 ± 0.00^d^
6.25	10.67 ± 0.67^ef^	09.67 ± 0.33^ef^	06.00 ± 0.00^d^	06.00 ± 0.00^d^
3.125	09.33 ± 0.88^f^	08.33 ± 0.33^f^	06.00 ± 0.00^d^	06.00 ± 0.00^d^

The values of mean zones of inhibition (MZI) are conveyed as mean ± std error of mean. Values having similar superscripts within a particular column are insignificantly distinct after one-way analysis of variance and Tukey's post hoc (*p* < 0.05).

**Table 2:** Minimum inhibitory concentration of *S. singueana* extract.

Concentration (mg/ml)		
Bacterial strain	S. *singueana* extract	Ciprofloxacin (*µ*g/ml)
*S. aureus*	1.30 ± 0.26^b^	0.16 ± 0.03^a^
*B. subtilis*	3.13 ± 0.00^b^	0.16 ± 0.03^a^
*S. typhi*	41.67 ± 8.33^a^	0.13 ± 0.03^a^
*E. coli*	20.83 ± 4.17^ab^	0.05 ± 0.00^a^

Values were conveyed as mean ± std error of mean. Values having similar superscript letters within a particular column are insignificantly distinct (*p* < 0.05) (two-sample *T*-test). *p* < 0.05.

**Table 3:** Comparison of MICs of the studied ethyl acetate extract and Ciprofloxacin.

	Minimum inhibition concentration (mg/ml)			
Treatment	*S. aureus*	*B. subtilis*	*S. typhi*	*E. coli*
Ciprofloxacin (*µ*g/ml)	0.16 ± 0.03^b^	0.16 ± 0.03^b^	0.13 ± 0.03^b^	0.05 ± 0.00^b^
*S. singueana* extract	1.30 ± 0.26^a^	3.13 ± 0.00^a^	41.67 ± 8.33^a^	20.83 ± 4.17^a^

Values were conveyed as mean±std error of mean. Values having similar superscript letters within a particular column are insignificantly distinct (*p* < 0.05) (two-sample *T*-test).

**Table 4:** Minimum bactericidal concentration of *S. singueana* extract.

Concentration (mg/ml)		
Bacterial strains	*S. singueana* extract	Ciprofloxacin (*µ*g/ml)
*S. aureus*	12.50 ± 0.00^c^	1.30 ± 0.26^a^
*B. subtilis*	41.67 ± 8.33^b^	0.65 ± 0.13^ab^
*S. typhi*	100.00 ± 0.00^a^	0.78 ± 0.00^ab^
*E. coli*	100.00 ± 0.00^a^	0.26 ± 0.06^b^

Values were conveyed as mean ± std error of mean. Values having similar superscript letters within a particular column are insignificantly distinct after one-way analysis of variance and Tukey's post hoc (*p* < 0.05).

**Table 5:** Comparison of MBC of the studied ethyl acetate extract and Ciprofloxacin.

Minimum bactericidal concentration (mg/ml)				
Treatment	*S. aureus*	*B. subtilis*	*S. typhi*	*E. coli*
Ciprofloxacin (*µ*g/ml)	1.30 ± 0.26^b^	0.65 ± 0.13^b^	0.78 ± 0.00^b^	0.26 ± 0.06^b^
*S. singueana* extract	12.50 ± 0.00^a^	41.67 ± 8.33^a^	100.00 ± 0.00^a^	100.00 ± 0.00^a^

Values were conveyed as mean ± std error of mean. Values having similar superscript letters within a particular column are insignificantly distinct (*p* < 0.05) (two-sample *T*-test).

**Table 6:** Quantitative phytochemical compound analysis in ethyl acetate extract of S. *singueana.*

RT (mins)	Compound	%abundance	MF	MW (g/mol)	Conc (*µ*g/g)	Chemical Class
38.78	*β*-Sitosterol	0.28	C_29_H_50_O	414.70	0.17 ± 0.00	Steroid
44.42	*γ*-Cyano-3-methyl-5,10-dihydrobenzo[f]indolizine	0.09	C_14_H_12_N_2_	208.26	0.06 ± 0.00	Heteroaromatic molecule
47.31	1(3H)*-*Isobenzofuranone, 6,7*–* dimethoxy-3-[2-(2-methoxyphenyl)-2-Oxoethyl]-	0.09	C_19_H_18_O_6_	342.30	0.05 ± 0.00	Volatile organic substances
46.71	1,2,3*-*Propatriol, 1-indol-4*-*yl (ether)	0.24	C_11_H_13_NO_3_	207.00	0.14 ± 0.00	Hydrocarbon
27.34	1,4-Dioxaspiro [4.5]decane*-*6-carboxylic acid, dimethylamide	2.53	C_11_H_19_NO_3_	213.27	1.51 ± 0.02	Cyclohexane
21.79	1-Cyclopentyleicosane	0.57	C_25_H_50_	350.70	0.34 ± 0.00	Hydrocarbon
27.06	1-Hexadecanol, 3,7,11,15-tetramethyl-	0.95	C_20_H_42_O	298.50	0.57 ± 0.01	Terpene alcohol
44.18	2-(Acetoxymethyl)-3-(methoxycarbonyl)biphenylene	0.72	C_17_H_14_O_4_	282.29	0.43 ± 0.01	Hydrocarbon
13.72	2-Cyclohexylpiperidine	0.05	C_11_H_21_N	167.29	0.03 ± 0.00	Alkaloid
46.38	2-Pyridinamine, N-(4, 5-dihydro-5-methyl-2-thiazolyl)-3-methyl-	0.15	C_10_H_13_N_3_S	207.29	0.09 ± 0.00	Aminopyridine
29.69	3,7,11,15*-*Tetramethyl*-*2*-*hexadecen-1-ol	5.57	C_20_H_40_O	296.50	3.32 ± 0.04	Terpene alcohol
21.51	4-Nonanol, 2,6,8-trimethyl-	0.56	C_12_H_26_O	186.33	0.33 ± 0.00	Fatty alcohol
49.10	5-(2-Oxo-6-phenyl-1,2-dihydropyrimidinyl-4)uracil	0.02	not found		0.01 ± 0.00	
25.51	7-Octenal, 3,7-dimethyl-	1.78	C_10_H_18_O	154.25	1.06 ± 0.01	Monoterpenoid
25.06	9-Octadecenoic acid, methyl ester, (E)-	2.05	C_19_H_36_O_2_	296.50	1.22 ± 0.02	Fatty acid methyl ester
28.18	9-Tricosene, (Z)-	4.53	C_23_H_46_	322.60	2.70 ± 0.04	Hydrocarbon
44.35	Acetamide, N-methyl-N-(2-phenylethyl)-	0.28	C_11_H_15_NO	177.24	0.17±0.00	Benzene derivative
03.39	Butane, 2-chloro-2-methyl-	0.33	C_5_H_11_Cl	106.59	0.20 ± 0.00	Alkyl chloride /chlorinated hydrocarbon
16.95	Cyclohexadecane, 1,2-diethyl-	0.52	C_20_H_40_	280.50	0.31 ± 0.00	Hydrocarbon
23.37	Decane, 3,8-dimethyl-	0.41	C_12_H_26_	170.33	0.24 ± 0.00	Hydrocarbon
25.88	Docosane	3.51	C_22_H_46_	310.60	2.09 ± 0.03	Hydrocarbon
19.40	Dodecane, 2,6,11-trimethyl*-*	0.68	C_15_H_32_	212.41	0.40 ± 0.01	Aliphatic alkane
20.16	Dodecanoic acid	4.17	C_12_H_24_O_2_	200.32	2.48 ± 0.03	Fatty acid
19.65	Dodecanoic acid, methyl ester	0.72	C_13_H_26_O_2_	214.34	0.43 ± 0.01	Fatty acid methyl ester
21.64	Eicosane (C20)	0.95	C_20_H_42_	282.50	0.57 ± 0.01	Hydrocarbon
30.38	Fumaric acid,4-methyl pent-2-yl tridecyl ester	1.28	C_23_H_42_O_4_	382.58	0.76 ± 0.01	Fatty acid ester
29.14	Hexacosane	3.44	C_26_H_54_	366.70	2.05 ± 0.03	Hydrocarbon
29.89	Hexadecane (C16)	4.50	C_16_H_34_	226.44	2.68 ± 0.04	Hydrocarbon
34.24	Hexadecane, 8-hexyl-8-pentyl-	1.82	C_27_H_56_	380.70	1.08 ± 0.01	Hydrocarbon
24.07	Hexadecanoic acid, ethyl ester	2.31	C_18_H_36_O_2_	284.50	1.38 ± 0.02	Fatty acid ester
24.33	Isopropyl hexadecanoate	1.01	C_19_H_38_O_2_	298.50	0.60 ± 0.01	Fatty acid ester
40.55	Lupan-3-ol	0.33	C_30_H_52_O	428.40	0.20 ± 0.00	Tritepenoid
23.46	Methyl hexadecanoate	1.60	C_17_H_34_O_2_	270.50	0.95 ± 0.01	Fatty acid methyl ester
18.88	Methyleugenol	0.52	C_11_H_14_O_2_	178.23	0.31 ± 0.00	Phenylpropanoid
23.84	n-Hexadecanoic acid	1.48	C_16_H_32_O_2_	256.42	0.88 ± 0.01	Fatty acid dervivative
26.73	Nonadecane (C19)	2.61	C_19_H_40_	268.50	1.50 ± 0.02	Hydrocarbon
30.72	Octacosane	3.41	C_28_H_58_	394.80	2.03 ± 0.03	Hydrocarbon
06.68	Oxazolidine, 2-ethyl-2-methyl-	0.03	C_6_H_13_NO	115.17	0.02 ± 0.00	Oxazoline compound
28.37	Pentacosane	3.63	C_25_H_52_	352.70	2.16 ± 0.03	Hydrocarbon
28.65	Phenol, 2,4*-*bis (1*-*methyl-1*-*phenylethyl)-	2.81	C_24_H_26_O	330.50	1.67 ± 0.02	Phenolic compound
25.21	Phytol	3.54	C_20_H_40_O	296.50	2.11 ± 0.03	Diterpenoid
26.08	Phytol acetate<E->	3.34	C_22_H_42_O_2_	338.60	1.99 ± 0.03	Diterpenoid
10.55	Pyrrolidine, 2-decyl-1-methyl-	0.07	C_17_H_35_N	253.50	0.04 ± 0.00	Cyclic secondary amine
31.07	Squalene	8.79	C_30_H_50_	410.70	5.24 ± .07	Triterpenoid
32.85	Tetracosane	8.48	C_24_H_50_	338.70	5.05 ± 0.03	Hydrocarbon
25.72	Tetratetracontane	2.40	C_44_H_90_	619.20	1.43 ± 0.02	Hydrocarbon
31.70	Tricosane	5.72	C_23_H_48_	324.60	3.41 ± 0.05	Hydrocarbon
21.19	Tridecane, 1-iodo-	1.03	C_13_H_27_I	310.26	0.61 ± 0.01	Iodo compound
24.21	Undecane, 5,5-dimethyl-	0.83	C_13_H_28_	184.36	0.50 ± 0.01	Hydrocarbon
35.06	Vitamin E	3.26	C_29_H_50_O_2_	430.70	1.94 ± 0.03	Tocopherol

Conc, concentration; Mins, minutes; MF, molecular formula; RT, retention time; MW, molecular weight.

## Data Availability

The data utilized to support the findings in this study are included in this article.
